# Pathway engineering of *Propionibacterium jensenii* for improved production of propionic acid

**DOI:** 10.1038/srep19963

**Published:** 2016-01-27

**Authors:** Long Liu, Ningzi Guan, Gexin Zhu, Jianghua Li, Hyun-dong Shin, Guocheng Du, Jian Chen

**Affiliations:** 1 Key Laboratory of Carbohydrate Chemistry and Biotechnology, Ministry of Education, Jiangnan University, Wuxi 214122, China; 2 Key Laboratory of Industrial Biotechnology, Ministry of Education, Jiangnan University, Wuxi 214122, China; 3 School of Chemical and Biomolecular Engineering, Georgia Institute of Technology, Atlanta 30332, USA

## Abstract

Propionic acid (PA) is an important chemical building block widely used in the food, pharmaceutical, and chemical industries. In our previous study, a shuttle vector was developed as a useful tool for engineering *Propionibacterium jensenii*, and two key enzymes—glycerol dehydrogenase and malate dehydrogenase—were overexpressed to improve PA titer. Here, we aimed to improve PA production further via the pathway engineering of *P. jensenii*. First, the phosphoenolpyruvate carboxylase gene (*ppc*) from *Klebsiella pneumoniae* was overexpressed to access the one-step synthesis of oxaloacetate directly from phosphoenolpyruvate without pyruvate as intermediate. Next, genes encoding lactate dehydrogenase (*ldh*) and pyruvate oxidase (*poxB*) were deleted to block the synthesis of the by-products lactic acid and acetic acid, respectively. Overexpression of *ppc* and deleting *ldh* improved PA titer from 26.95 ± 1.21 g·L^−1^ to 33.21 ± 1.92 g·L^−1^ and 30.50 ± 1.63 g·L^−1^, whereas *poxB* deletion decreased it. The influence of this pathway engineering on gene transcription, enzyme expression, NADH/NAD^+^ ratio, and metabolite concentration was also investigated. Finally, PA production in *P. jensenii* with *ppc* overexpression as well as *ldh* deletion was investigated, which resulted in further increases in PA titer to 34.93 ± 2.99 g·L^−1^ in a fed-batch culture.

According to the US Department of Energy, propionic acid (PA) is one of the top 30 candidate platform chemicals^1^. PA has multiple applications in the food, pharmaceutical, and chemical industries^2^. It is a key building block in the organic synthesis of cellulose fiber, perfume, paint, and herbicides and is widely used as an effective mold inhibitor in foods and feedstuffs^3^. PA is mainly produced through petrochemical synthesis via the hydrocarboxylation of ethylene. Given the increasing concerns about environmental pollution and petroleum shortages, bio-based PA production by propionibacteria from renewable resources has generated ongoing interest^4^. Propionibacteria have long been used to synthesize PA owing to their tenacious vitality, high yields, capacity to use a wide variety of substrates, and antimicrobial properties^5^.

Extensive studies have been carried out to improve PA yield and productivity in propionibacteria. A range of carbon sources including glucose^6^, xylose^7^, lactose^8^, sucrose^9^, lactic acid^10^, and maltose^11^ and many renewable and low-cost substrates such as hemicellulose^12^, sweet whey permeate^13^, corn meal^14^, and cane molasses^15^ can be used by propionibacteria to synthetize PA. In addition, fed-batch culture^16^, extractive fermentation^17^, cell immobilization^11^, pH-shift control^18^, oxidoreduction potential-shift control^19^, and plant fibrous-bed bioreactor^20^ techniques have been developed to improve PA production.

With improvements in the availability of genomic information and advances in synthetic biology, metabolic engineering of propionibacteria to enhance PA production has attracted great interest. However, strong restriction-modification systems have hampered the construction of molecular biology tools, and the metabolic engineering of propionibacteria has progressed slowly^5^. Until now, PA titers have been improved by overexpressing glycerol dehydrogenase, malate dehydrogenase, and fumarate hydratase in *Propionibacterium jensenii*^21^, phosphoenolpyruvate carboxylase (PPC) in *Propionibacterium freudenreichii*^22^, and propionyl-coenzyme A (CoA):succinate CoA transferase in *Propionibacterium shermanii*^23^. In our previous studies, the shuttle vector pZGX04 was constructed as an effective tool for engineering *P. jensenii*^24^, and PA synthesis was enhanced by overexpressing glycerol dehydrogenase and malate dehydrogenase^21^,^24^.

As shown in Fig. 1, the PA synthesis pathway in *P. jensenii* pyruvate carboxylase converts pyruvate to oxaloacetate, and PA is generated through seven intermediates, including malate, succinyl-CoA, and propionyl-CoA. Furthermore, lactate and acetate are by-products of pyruvate generation. PPC, although not found in propionibacteria, converts phosphoenolpyruvate to oxaloacetate by fixing CO_2_ in some microorganisms^22^. Thus, carbon flux contributes to the earlier accumulation of PA without the reaction proceeding through pyruvate, and the carbon flux to lactate and acetate is eliminated at the same time. Several studies have shown that improvements in PPC activity enhance the production of organic acids such as succinate^25^ and fumarate^26^ in *Escherichia coli*.

In this study, the gene encoding PPC (*pp*c) from *Klebsiella pneumoniae* was overexpressed in *P. jensenii* by pZGX04, while the genes encoding lactate dehydrogenase (*ldh*) and pyruvate oxidase (*poxB*) were deleted from *P. jensenii* by pUC18. The effects of these genetic manipulations on cell growth, metabolic levels, and PA production were then investigated.

## Results

### Expression of *ppc* and deletion of *ldh* and *poxB* in *P. jensenii* ATCC 4868

The genome of *K. pneumoniae* was used as the template for *ppc* amplification because detailed genome information for *P. jensenii* is lacking and the genes of *K. pneumoniae* have been successfully expressed^20^. The expression vector pZGX04-ppc was constructed based on pZGX04 and transformed into *E. coli* JM110 for demethylation to improve the transformation efficiency of recombinant plasmids by eliminating the influence of the restriction-modification system in *P. jensenii*^23^. The engineered strain *P. jensenii* (pZGX04-ppc) was obtained by transforming the demethylated pZGX04-ppc into *P. jensenii* ATCC 4868. pUC18 was used as suicide plasmid for gene deletion in *P. jensenii* because it is unable to replicate and be expressed in propionibacteria. Similarly, the LDH deletion vector pUC18-ldh-cm and POXB deletion vector pUC18-poxB-cm were transformed into *P. jensenii* ATCC 4868 after demethylation via *E. coli* JM110, and the engineered strains *P. jensenii*-Δldh and *P. jensenii*-ΔpoxB were constructed through homologous recombination.

### PA production via fed-batch culture of the engineered *P. jensenii*

Fed-batch culture of *P. jensenii* ATCC 4868, *P. jensenii* (pZGX04-ppc), *P. jensenii*-Δldh and *P. jensenii*-ΔpoxB in 3-L bioreactors was performed under optimized culture conditions with a pH-shift control strategy. The parameters for PA production are listed in Table 1, and the fermentation kinetics are shown in Fig. 2. The maximum PA concentration of *P. jensenii* ATCC 4868 reached 26.95 ± 1.21 g·L^−1^ at 228 h, whereas the dry cell weight (DCW) was 3.55 ± 0.19 g·L^−1^. The main by-products were 2.86 ± 0.17 g·L^−1^ lactate and 2.44 ± 0.14 g·L^−1^ acetate. Compared with that of the wild type, the PA production of *P. jensenii* (pZGX04-ppc) (33.21 ± 1.92 g·L^−1^) and *P. jensenii*-Δldh (30.50 ± 1.63 g·L^−1^) increased by 23.23% and 13.17%, respectively. However, *P. jensenii*-ΔpoxB produced only 20.12 ± 1.04 g·L^−1^ PA. The acetate production of *P. jensenii* (pZGX04-ppc) and lactate production of *P. jensenii*-Δldh decreased significantly and only small amounts of acetate and lactate were produced by *P. jensenii*-ΔpoxB.

The cell growth of *P. jensenii* (pZGX04-ppc), *P. jensenii*-Δldh, and *P. jensenii*-ΔpoxB was lower than that of *P. jensenii* ATCC 4868, and DCW dropped to 3.38 ± 0.18 g·L^−1^, 3.31 ± 0.11 g·L^−1^, and 1.99 ± 0.008 g·L^−1^, respectively. As a result, the specific PA titer increased from 7.59 ± 0.46 g·L^−1^·g^−1^ DCW in *P. jensenii* ATCC 4868 to 9.83 ± 0.55 g·L^−1^·g^−1^ DCW, 9.08 ± 0.37 g·L^−1^·g^−1^ DCW, and 10.11 ± 0.48 g·L^−1^·g^−1^ DCW in *P. jensenii* (pZGX04-ppc), *P. jensenii*-Δldh, and *P. jensenii*-ΔpoxB, respectively. Moreover, the fermentation period of *P. jensenii*-Δldh extended to 240 h. However, the production intensity of *P. jensenii* (pZGX04-ppc) and *P. jensenii*-Δldh increased to 0.146 g·L^−1^·h^−1^ and 0.127 g·L^−1^·h^−1^, respectively, compared with 0.118 g·L^−1^·h^−1^ in the wild type.

### Effects of gene expression and deletion on intracellular NADH/NAD^+^ ratio

Because *ppc* overexpression and *ldh* deletion have been proved effective for improving PA production, further investigation of *P. jensenii* (pZGX04-ppc) and *P. jensenii*-Δldh was undertaken. To verify the transcription of *ppc* and *ldh* in the corresponding engineered strains, we performed reverse transcription-qPCR, which introduced the 16S ribosomal RNA-encoding gene as the reference gene for normalization. As shown in Fig. 3A, compared with those in *P. jensenii* ATCC 4868, the transcription level of *ppc* in *P. jensenii* (pZGX04-ppc) increased 24.54 ± 1.34-fold, whereas that of *ldh* in *P. jensenii*-Δldh decreased 11.29 ± 0.91-fold. These results indicating that pZGX04-ppc and pUC18-ldh-cm were successfully transformed into in *P. jensenii*, *ppc* was transcribed to messenger RNA, and *ldh* was knocked out.

The specific activities of PPC and LDH in *P. jensenii* ATCC 4868 and the corresponding engineered strains were compared (Fig. 3B). The specific activity of PPC was 9.72 ± 0.44 U·g^−1^ DCW in *P. jensenii* (pZGX04-ppc) but was not detected in *P. jensenii* ATCC 4868. The specific activity of LDH in *P. jensenii*-Δldh was 4.67 times lower than that in *P. jensenii* ATCC 4868. These results further demonstrated that the recombinant plasmids are effective for metabolic regulation in *P. jensenii*.

The intracellular NADH/NAD^+^ ratios of *P. jensenii* ATCC 4868, *P. jensenii* (pZGX04-ppc), and *P. jensenii*-Δldh at the middle exponential, later exponential, and stationary phases were analyzed. As shown in Fig. 3C, the intracellular NADH/NAD^+^ ratios of the three strains decreased with the length of fermentation. Although the intracellular NADH/NAD^+^ ratio in *P. jensenii* (pZGX04-ppc) (3.8 ± 0.30) was similar to that in *P. jensenii* ATCC 4868 (3.9 ± 0.36) at the middle exponential phase, it dropped rapidly during the later exponential phase (2.5 ± 0.31) and stationary phase (1.3 ± 0.22) of the culture and was significantly lower than those in *P. jensenii* ATCC 4868 (3.0 ± 0.28 and 1.6 ± 0.19, respectively). However, the intracellular NADH/NAD^+^ ratios in *P. jensenii*-Δldh at all three phases were always higher than those in *P. jensenii* ATCC 4868.

### Changes in intracellular metabolites

To investigate the influence of expressing *ppc* and knocking out *ldh* on the intracellular metabolites of *P. jensenii*, we detected the intercellular metabolites of *P. jensenii* ATCC 4868, *P. jensenii* (pZGX04-ppc), and *P. jensenii*-Δldh at the exponential and stationary phases of fed-batch cultures. A total of nine key metabolites were selected for analysis: glycerate-3-phosphate, phosphoenolpyruvate, pyruvate, lactate, acetyl-CoA, oxaloacetate, citrate, malate, and propionyl-CoA. The content of these metabolites in the engineered strains relative to those of *P. jensenii* ATCC 4868 are shown in Fig. 4. PPC expression decreased phosphoenolpyruvate and increased oxaloacetate. As a result, the intercellular concentrations of glycerate-3-phosphate, pyruvate, lactate, and acetyl-CoA decreased. In particular, the level of pyruvate decreased significantly with the increase in PA synthesis, which was less than one-fifth that of the wild type at the stationary phase. Relatively, intercellular levels of citrate, malate, and propionyl-CoA increased, among which propionyl-CoA content increased 1.2- to 1.4-fold.

The deletion of *ldh* blocked the accumulation of lactate directly. At the exponential phase, the intercellular concentration of propionyl-CoA in *P. jensenii*-Δldh was substantially higher than that in the wild type—an increase of 89.0%. Simultaneously, the content of phosphoenolpyruvate, pyruvate, acetyl-CoA, oxaloacetate, and malate increased. Acetyl-CoA level increased by 75.1% in *P. jensenii*-Δldh at the stationary phase; however, the levels of other metabolites changed only slightly.

### PA production in a fed-batch culture of *P. jensenii*-Δldh (pZGX04-ppc)

Because the expression of *ppc* and deletion of *ldh* enhanced PA synthesis in *P. jensenii*, we constructed the engineered strain *P. jensenii*-Δldh (pZGX04-ppc), in which *ppc* was overexpressed and *ldh* was knocked out. The result of a fed-bath culture for PA production by *P. jensenii*-Δldh (pZGX04-ppc) is shown in Fig. 5. Compared with that of *P. jensenii* ATCC 4868, *P. jensenii* (pZGX04-ppc), and *P. jensenii*-Δldh, the PA production of *P. jensenii*-Δldh (pZGX04-ppc) (34.93 ± 2.99 g·L^−1^) increased of 29.61%, 5.18%, and 14.52%, respectively. The DCW of *P. jensenii*-Δldh (pZGX04-ppc) decreased to 3.19 ± 0.39 g·L^−1^, and the fermentation period extended to 240 h. However, the production intensity of the engineered strain (0.146 g·L^−1^·h^−1^) was still improved.

## Discussion

*P. jensenii* is a species of dairy propionibacteria that has been used to produce PA via the dicarboxylic acid pathway with acetate and lactate as by-products^21^,^27^. Low yield, low productivity, and poor purity caused by feedback inhibition and high by-product accumulation have seriously restricted the industrialization of microbial PA production by propionibacteria^23^. Various strategies have been proposed to improve the fermentation process and enhance PA synthesis^3^. However, the majority of these efforts involve strain evolution^28^,^29^ and fermentation optimization^16^,^19^. Metabolic engineering of propionibacteria has progressed slowly due to the lack of genome information, the shortage of available gene manipulation tools, the difficulties of transforming gram-positive bacteria, and the high GC content of genomes^5^. Recently, the genomes of several propionibacteria were fully sequenced, and in turn, the synthetic pathways of PA were elucidated^30^,^31^,^32^. The availability of this information provided the rationale and basis for the metabolic engineering of propionibacteria for improved PA production.

pZGX04, a *Propionibacterium*-*E. coli* shuttle plasmid, is the only reported gene expression vector available for *P. jensenii*^24^. A fermentation optimization strategy was developed for engineered *P. jensenii* in our recent study and integrates the use of a fed-batch culture with pH-shift control^18^. Using this strategy, we overexpressed *ppc*, knocked out *ldh* and *poxB* in *P. jensenii* ATCC 4868 by constructing *P. jensenii* (pZGX04-ppc), *P. jensenii*-Δldh and *P. jensenii*-ΔpoxB, respectively, and further enhanced PA production.

**Figure 1:**
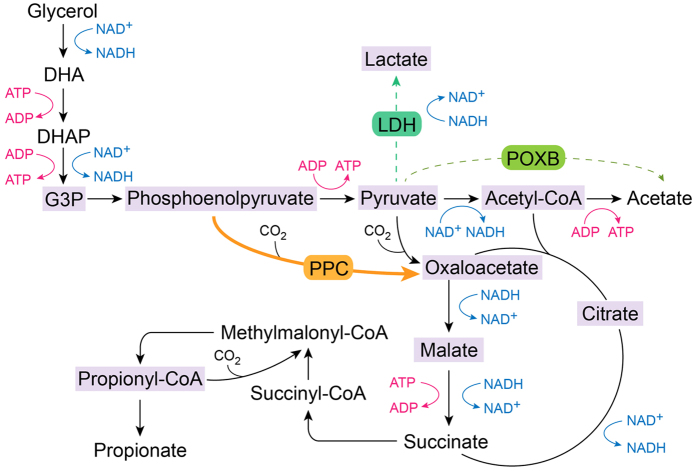
Biosynthetic pathways of propionic acid, lactic acid, and acetic acid from glycerol in *Propionibacterium*. DHA, dehydroxyacetone; DHAP, dihydroxyacetone phosphate. The pathway introduced was indicated with bold line, while the pathway deleted was indicated with dotted line.

**Figure 2:**
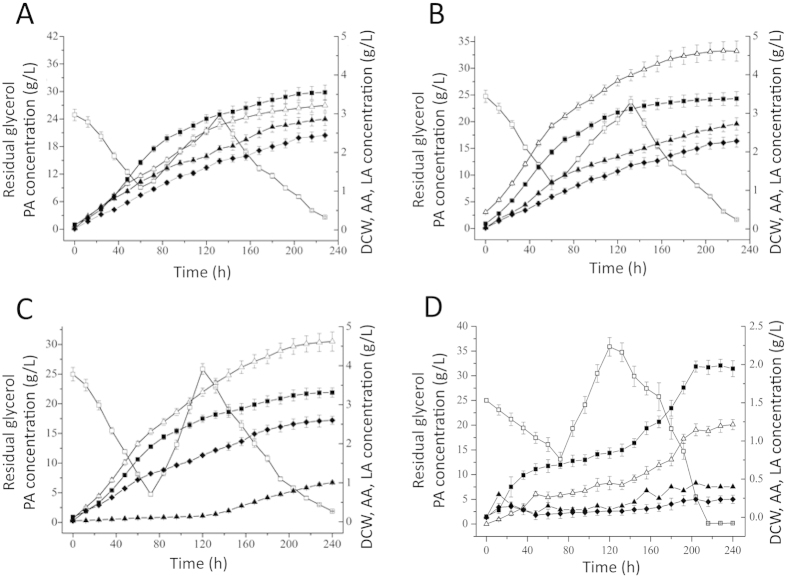
Fed-batch culture kinetics of propionic acid production from glycerol with *Propionibacterium jensen*ii ATCC 4868 wild type and the engineered strains. (**A**), *P. jensenii* ATCC 4868; (**B**), *P. jensenii* (pZGX04-ppc); (**C**), *P. jensenii*-Δldh; (**D**), *P. jensenii*-ΔpoxB. The culture temperature and agitation speed were maintained at 32 ^o^C and 200 rpm, respectively. pH was controlled at 5.9 for 0–36 h, and was shifted to 6.5 after 36 h via automatic addition of Ca(OH)_2_ (10% [w/v]). Concentrated glycerol (300 g·L^−1^) was fed at a constant rate of 3.33 mL·h^−1^ between 60–132 h during culture process. Δ, propionic acid (PA); ◻, residual glycerol; ◼, dry cell weight (DCW); ♦, acetic acid (AA); ▲, lactic acid (LA).

**Figure 3:**
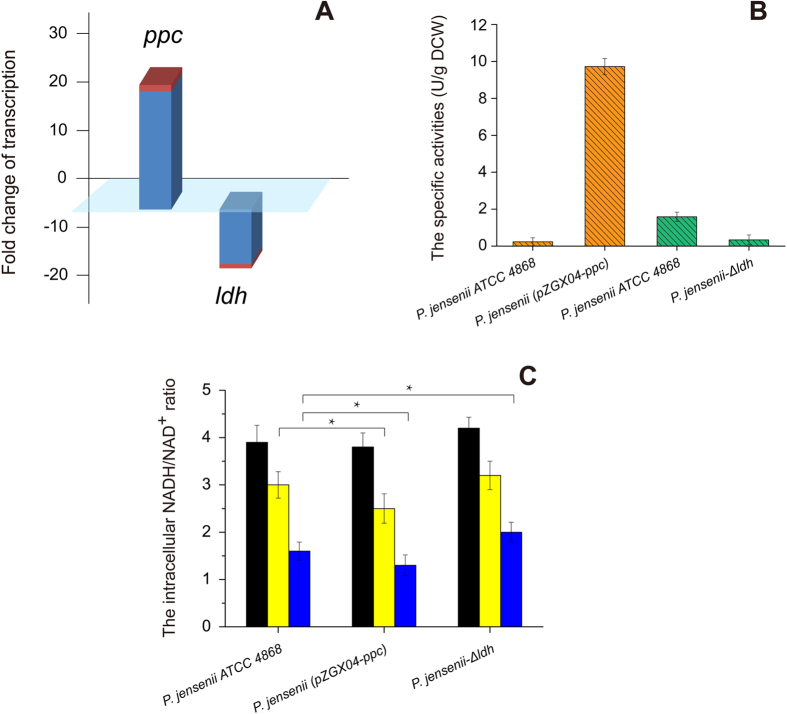
(**A**) The transcription level of ppc and ldh in *P. jensenii* (pZGX04-ppc) and *P. jensenii*-Δldh relative to those in *P. jensenii* ATCC 4868; (**B**) Specific enzyme activities of phosphoenolpyruvate carboxylase (Orange) and lactate dehydrogenase (Green) in *P. jensenii* ATCC 4868 and the engineered strains; (**C**) NADH/NAD^+^ ratio of *P. jensenii* ATCC 4868 and the engineered strains in different phases. Black bar, middle exponential phase; yellow bar, later exponential phase; blue bar, stationary phase; *, significant difference (*P* ≤ 0.05)

**Figure 4:**
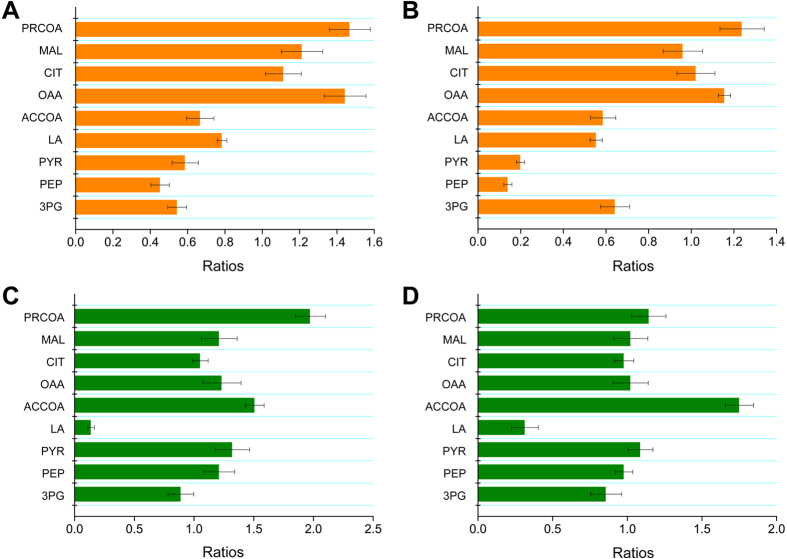
The contents of intracellular metabolites in the engineered strains relative to *P. jensenii* ATCC 4868. PRCOA, propionyl-CoA; MAL, malate; CIT, citrate; OAA, oxaloacetate; ACCOA, acetyl-CoA; LA, lactate; PYR, pyruvate; PEP, phosphoenolpyruvate; 3PG, glycerate 3-phosphate. (**A**) The contents of intracellular metabolites in *P. jensenii* (pZGX04-ppc) relative to *P. jensenii* ATCC 4868 at exponential phase; (**B**) The contents of intracellular metabolites in *P. jensenii* (pZGX04-ppc) relative to *P. jensenii* ATCC 4868 at stationary phase; (**C**) The contents of intracellular metabolites in *P. jensenii*-Δldh relative to *P. jensenii* ATCC 4868 at exponential phase; (**D**) The contents of intracellular metabolites in *P. jensenii*-Δldh relative to *P. jensenii* ATCC 4868 at stationary phase.

**Figure 5:**
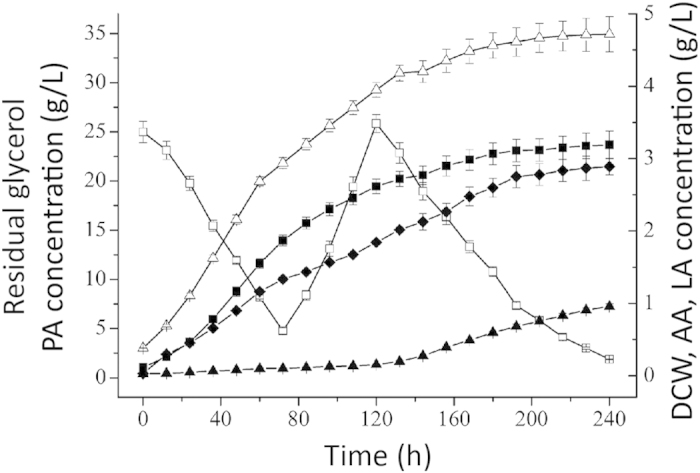
Fed-batch culture kinetics of propionic acid production from glycerol with *P. jensenii*-Δldh (pZGX04-ppc). Δ, propionic acid (PA); ◻, residual glycerol; ◼, dry cell weight (DCW); ♦, acetic acid (AA); ▲, lactic acid (LA).

Pyruvate is a key metabolic node in the synthetic pathway of PA in propionibacteria^33^. A total of three branch pathways are derived from pyruvate: acetyl-CoA, which enters the tricarboxylic acid cycle or generates acetate; lactate formed by the catalysis of LDH; and oxaloacetate, which ultimately leads to PA (see Fig. 1). Methylmalonyl-CoA carboxyltransferase and pyruvate carboxylase convert pyruvate to oxaloacetate in certain propionibacteria through the transfer of carbon from methylmalonyl-CoA to pyruvate and the fixing of CO_2_, respectively. Furthermore, PPC converts phosphoenolpyruvate to oxaloacetate via CO_2_ fixation in some microorganisms, although not in propionibacteria^22^. Therefore, in the engineered strains, the carbon flux flowed to oxaloacetate from phosphoenolpyruvate earlier, bypassing pyruvate, and the metabolic flux to acetyl-CoA and lactate was reduced as pyruvate was generated in smaller amounts (see Fig. 4A,B). As a result, the flux to oxaloacetate increased and the metabolism of the tricarboxylic acid cycle and PA synthesis was enhanced, as suggested by the improved levels of citrate, malate, and propionyl-CoA. In addition, the flow of *P. jensenii* (pZGX04-ppc) was more focused on the PA synthetic pathway, as demonstrated by the higher increases in malate and propionyl-CoA. Therefore, PA production was enhanced by overexpressing *ppc* in *P. jensenii* and decreasing the accumulation of by-products (acetate and lactate; see Fig. 2B).

NADH and NAD^+^ play crucial roles in the intracellular chemical reactions and metabolism of microbial cells^34^. NADH is consumed along the metabolic pathway from pyruvate to PA, which changes the NADH/NAD^+^ ratio^19^. That is, the NADH/NAD^+^ ratio is an indicator of the synthetic efficiency of *P. jensenii* for PA. Furthermore, it has been demonstrated in *E. coli* that a reducing intracellular environment is necessary for key cellular functions^35^. Compared with *P. jensenii* ATCC 4868, *P. jensenii* (pZGX04-ppc) had lower NADH/NAD^+^ ratios at the later exponential phase and stationary phase, which indicated that the introduction of PPC accelerated PA synthesis in the late fermentation stage and consumed more NADH. These results coincided well with the higher PA production of *P. jensenii* (pZGX04-ppc).

Because lactate is one of the main by-products of glycerol metabolism in the fed-batch culture of *P. jensenii*, *ldh* was knocked out in *P. jensenii* ATCC 4868. Compared with that of *P. jensenii* ATCC 4868, the NADH/NAD^+^ ratio of *P. jensenii*-Δldh was higher because NADH is consumed during lactate synthesis from pyruvate. The activities of many intracellular enzymes were influenced by the variation in NADH/NAD^+^ ratios, and thus the distribution of intracellular metabolic flow was influenced^36^. Therefore, the higher reducibility maintained in *P. jensenii*-Δldh has a negative effect on microbial activity, which results in slow cell growth and an extended fermentation period. Simultaneously, the lack of a lactate pathway leads to an increase in metabolic flow from pyruvate to PA and acetate, which was shown by the increased concentrations of acetyl-CoA, oxaloacetate, malate, and propionyl-CoA in *P. jensenii*-Δldh.

The growth of *P. jensenii* was strongly inhibited as *poxB* deleted. It is supposed that low accumulation of acetate affects the metabolism of acetyl phosphate, which has been proved to be a regulator of signal transduction in the two-component regulatory systems in some bacteria^37^. Presecan-Siedel *et al.* also found that *Bacillus subtilis* did not grow when acetate kinase was inactivated^38^. Therefore, the unnormal metabolism of acetyl phosphate may be responsible for the growth defect of *P. jensenii*-ΔpoxB.

In this study, we enhanced PA production in *P. jensenii* by constructing the engineered strains *P. jensenii* (pZGX04-ppc) and *P. jensenii*-Δldh. The overexpression of *ppc* decreased the accumulation of by-products and accelerated PA biosynthesis by adjusting the distribution of metabolic flow, and the production and productivity of PA in *P. jensenii* (pZGX04-ppc) increased to 33.21 ± 1.92 g·L^−1^ and 7.59 ± 0.46 g·L^−1^·g^−1^ DCW, respectively. The deletion of *ldh* altered the redox state of the cells and reduced the cell growth rate, and PA production and productivity in *P. jensenii*-Δldh increased to 30.50 ± 1.63 g·L^−1^ and 9.08 ± 0.37 g·L^−1^·g^−1^ DCW, respectively. Furthermore, by expressing *ppc* and deleting *ldh* simultaneously, we increased the production of PA to 34.93 ± 2.99 g·L^−1^, an increase of 29.61% over production by wild-type *P. jensenii* ATCC 4868. Since overexpression of glycerol dehydrogenase, malate dehydrogenase, fumarate hydratase, and PPC, and deletion of *ldh* have been proved to be effective on improving PA production of *P. jensenii*^21^, further studies should be conducted to investigate the combinatorial effect of them on PA production in the future work.

## Materials and Methods

### Strains, plasmids, and culture conditions

*P. jensenii* ATCC 4868 and *K. pneumoniae* subsp. *pneumoniae* ATCC 12657 were purchased from the American Type Culture Collection (ATCC) and used as templates to amplify genes or as hosts for gene expression and deletion. *E. coli* JM110 (Stratagene, La Jolla, CA) was used for plasmid cloning and maintenance. pUC18 (TaKaRa, Dalian, China) was used as suicide plasmid to knock out genes in *P. jensenii*, and the shuttle vector pZGX04 was constructed in our previous study^24^. pRS303 was maintained in our laboratory to amplify the hygromycin B resistance gene (*hygB*). *P. jensenii* was cultured anaerobically at 32 °C in sodium lactate broth medium (10 g·L^−1^ yeast extract, 10 g·L^−1^ tryptic soy broth, and 10 g·L^−1^ sodium lactate), and *E. coli* and *K. pneumoniae* were grown at 37 °C in Luria-Bertani medium (10 g·L^−1^ NaCl, 10 g·L^−1^ peptone, and 5 g·L^−1^ yeast extract) with a shaking speed of 220 rpm. When necessary, 10 μg·mL^−1^ chloramphenicol and 50 μg·mL^−1^ hygromycin B were added to the *P. jensenii* cultures, and 100 μg·mL^−1^ ampicillin was added to the *E. coli* cultures.

### Construction of plasmids and recombinant strains

The genomic DNA of *K. pneumoniae* and *P. jensenii* were isolated with an E.Z.N.A.^TM^ Bacterial DNA Kit (Omega Bio-Tek, Doraville, GA, USA). *ppc* was amplified via polymerase chain reaction (PCR) from the genome of *K. pneumoniae*, and the PCR products were purified with a TaKaRa MiniBEST Agarose Gel DNA Extraction Kit (TaKaRa). The expression vector pZGX04-ppc was constructed by replacing *orf2* in pZGX04 with *ppc*. The front and back sequences flanking *ldh* and *poxB* were amplified from the genome of *P. jensenii*, and the chloramphenicol resistance gene (*cm*) and *hygB* were amplified from pZGX04 and pRS303, respectively. The *ldh*/*poxB* deletion frame was constructed by fusing the three fragments via PCR and inserting them into pUC18 to obtain the deletion vectors pUC18-ldh-cm and pUC18-poxB-hygB. The primers used for PCR are listed in Table 2.

pZGX04-ppc, pUC18-ldh-cm, and pUC18-poxB-hygB were first transformed into *E. coli* JM110 for demethylation and then transformed into *P. jensenii* ATCC 4868 to construct *P. jensenii* (pZGX04-ppc), *P. jensenii*-Δldh, and *P. jensenii*-ΔpoxB^24^. The construction of *P. jensenii*-Δldh (pZGX04-ppc) was carried out by transforming pZGX04-ppc into *P. jensenii*-Δldh with the same method. The deletion of *ldh*/*poxB* and expression of *ppc* were verified through colony PCR and sequencing. The expression of *ppc* was controlled by the original promoter of *orf2* in pZGX04.

### Microbial production of PA by the engineered *P. jensenii*

*P. jensenii* was activated in 100-mL anaerobic jars containing 100 mL seed medium (10 g·L^−1^ yeast extract, 5 g·L^−1^ Trypticase soy broth, 2.5 g·L^−1^ K_2_HPO_4_, and 1.5 g·L^−1^ KH_2_PO_4_) supplemented with 10 μg·mL^−1^ chloramphenicol. The inoculum was prepared in 250-mL anaerobic jars containing 200 mL of the same medium. The jars were sealed with butyl rubber caps and incubated at 32 °C for 48 h under stilling culture conditions. Fed-batch fermentations were carried out in a 3-L bioreactor (Eppendorf, Hamburg, Germany) containing 2 L culture medium (25 g·L^−1^ glycerol, 10 g·L^−1^ yeast extract, 5 g·L^−1^ peptone, 2.5 g·L^−1^ K_2_HPO_4_, 1.5 g·L^−1^ KH_2_PO_4_, and 8 mg·L^−1^ CoCl_2_) according to a previously developed protocol, which combined a two-stage pH-control strategy controlled by Ca(OH)_2_ (10%, w/v) with constant feeding of concentrated glycerol (300 g·L^−1^) between 60 and 132 h during culture^18^.

Ten-milliliter aliquots were removed from the fermenter every 12 h to measure the cell density and concentrations of PA, lactic acid, acetic acid, and glycerol according to previously published methods^16^. The influence of sampling volume was negligible, and the change in volume caused by Ca(OH)_2_ solution addition was removed for calculations.

### Reverse transcription-quantitative PCR (RT-qPCR)

Cells were harvested in the exponential phase when the optical density at 600 nm reached 0.6–0.7 in sodium lactate broth medium. Total RNA was isolated by using an RNAprep pure Cell/Bacteria kit (TIANGEN, Beijing, China). Complementary DNA was obtained by using a PrimeScript II first-strand complementary DNA synthesis kit (TaKaRa), and qPCR was conducted with SYBR Premix Ex Taq (TaKaRa) on a LightCycler 2.0 system (Roche, Basel, Switzerland). The fold changes were determined by the 2^−ΔΔCt^ method normalized to the 16S rRNA gene.

### PPC and LDH activity assays

Samples of *P. jensenii* ATCC 4868, *P. jensenii* (pZGX04-ppc), and *P. jensenii*-Δldh were collected at the exponential phase (48 h) and centrifuged at 7,000 x *g* for 10 min. The PPC activity of the cells was measured according to the method of Ammar *et al.*^22^, and the LDH activity of the cells was measured according to the method of Zheng *et al.*^39^.

### Determination of intracellular metabolites

Cells were sampled and quenched at the middle exponential phase, later exponential phase, and stationary phases for intracellular metabolite detection. The extraction and detection of metabolites were performed by using the procedures described in our previous study^19^.

### Statistical analysis

Unless otherwise noted, at least three independent experiments were performed for each conclusion, and the average values with standard errors are reported. Student’s *t*-test analysis was performed with SPSS 12.0 software (SPSS Inc., Chicago, USA) to investigate statistical differences. *P* values of less than 0.05 were considered statistically significant.

## Additional Information

**How to cite this article**: Liu, L. *et al.* Pathway engineering of *Propionibacterium jensenii* for improved production of propionic acid. *Sci. Rep.* **6**, 19963; doi: 10.1038/srep19963 (2016).

## Figures and Tables

**Table 1:** Analysis of fed-batch culture parameters for PA production with different strains.

Strain	Fermentation period (h)	DCW (g·L^−1^)	PA titer (g·L^−1^)	LA titer (g·L^−1^)	AA titer (g·L^−1^)	Specific PA titer (g·L^−1^·g^−1^ DCW)	PA productivity (g·L^−1^·h^−1^)
*P. jensenii* ATCC 4868	228	3.55 ± 0.19	26.95 ± 1.21	2.86 ± 0.17	2.44 ± 0.14	7.59 ± 0.46	0.118 ± 0.006
*P. jensenii* (pZGX04-ppc)	228	3.38 ± 0.18	33.21 ± 1.92	2.85 ± 0.12	2.24 ± 0.13	9.83 ± 0.55	0.146 ± 0.008
*P. jensenii*-Δldh	240	3.31 ± 0.11	30.50 ± 1.63	1.01 ± 0.08	2.60 ± 0.21	9.08 ± 0.37	0.127 ± 0.007
*P. jensenii*-ΔpoxB	240	1.99 ± 0.08	20.10 ± 1.04	0.45 ± 0.01	0.23 ± 0.06	10.11 ± 0.48	0.084 ± 0.004

**Table 2:** Primers used for plasmid construction.

Primer	Nucleotide sequence (5′-3′)
ppc-F	ATGAGATGGGGTGTCTGGG
ppc-R	TTAACCGGTGTTACGCATACC
pZGX04-orf2-F	CGGGGTACCTGGACGATTGAGTACACCGAC
pZGX04-orf2-R	CCATCGATCATCACGCACCTCCTGACTAC
ldh-left-F	ATGAGGTCGAACAAGCTGGTC
ldh-left-R	TGCGTGTCAGCGGTAGGTCGTCGTGTCGAGCGGATTGGT
cm-F	CCTACCGCTGACACGCAACCCACACCCGAACATGTCGCGT
cm-R	CCTTTCCCGCGTTGGTGGGGTCAATTGGCCTCCGCCGC
ldh-right-F	CCCCACCAACGCGGGAAAGGGACTCACGGTGGGGGGCTACC
ldh-right-R	CTACCGGACGCGCGGGGT
poxB-left-F	ACTGCCAGGATCACCTGG
poxB -left-R	GAGTTCAGGCTTTTTACCCATTGATTACCTCCGGATCCA
hygB-F	TGGATCCGGAGGTAATCAATGGGTAAAAAGCCTGAACTC
hygB-R	TTGAACACCACCTTGCTCATTTATTCCTTTGCCCTCGG
poxB-right-F	CCGAGGGCAAAGGAATAAATGAGCAAGGTGGTGTTCAA
poxB -right-R	GTCCGAAACCACTGCCG
